# Metastatic Epithelial-Myoepithelial Carcinoma in a Female Presenting with Neck Mass and Lytic Lesion in Acetabulum: A Diagnostic Challenge on Cytology

**DOI:** 10.5146/tjpath.2020.01501

**Published:** 2021-01-15

**Authors:** Parikshaa Gupta, Arvind Rajwanshi, Nandita Kakkar

**Affiliations:** Department of Cytology and Gynecological Pathology, Postgraduate Institute of Medical Education and Research, Chandigarh, India; Department of Histopathology, Postgraduate Institute of Medical Education and Research, Chandigarh, India

**Keywords:** Epithelial-myoepithelial carcinoma, Salivary gland neoplasms, Fine needle aspiration cytology, Cytodiagnosis, GATA3

## Abstract

Epithelial-myoepithelial carcinoma (EMC) is a rare, low-grade, malignant salivary neoplasm. Establishing an accurate cytological diagnosis is often challenging owing to its rarity, bland cytologic appearance and variable representation of cell populations in the smears. The diagnostic struggle is more so when the aspiration is from a metastatic site with an unknown primary, as in such cases the list of differential diagnoses expands further. A 58-year-old female presented with a low-back pain from last one month. On examination, she also had a level III, right cervical swelling for the last 20 years. Radiology revealed a lytic lesion in the left acetabulum. She had undergone surgery 35 years ago for a right-sided upper neck swelling, the medical records of which were not available. Fine needle aspiration (FNA) from the cervical swelling was performed. The smears were cellular and showed predominantly dispersed, round to polygonal tumor cells with mild pleomorphism, eccentric nuclei, coarse chromatin, occasional nucleoli and moderate cytoplasm with some showing vacuolations. The cell-block section revealed tumor cells arranged in the form of tubules lined by dual layer of tumor cells without any chondromyxoid stroma. On immunocytochemistry, the luminal cells showed positivity for CK7 (epithelial marker) and the abluminal cells showed positivity for p63 (myoepithelial marker). Based on these features, a final diagnosis of metastatic epithelial-myoepithelial carcinoma was rendered. The present report highlights the characteristic cytomorphological and immunocytochemical features of EMC and reiterates the diagnostic accuracy of FNAC for diagnosis of such challenging cases.

## INTRODUCTION

Epithelial-myoepithelial carcinoma (EMC) is a rare low-grade malignant neoplasm of the salivary glands. It mainly involves the major salivary glands, the most common being the parotid. EMCs account for less than 1% of all the salivary gland neoplasms and occur mostly in elderly females (1). As the name suggests, the tumor is composed of two different cell types, the epithelial and the myoepithelial cells. Although rare, the characteristic histopathological features are well-described with the tumor being composed of tubules having luminal ductal epithelial cells with scant eosinophilic cytoplasm and abluminal myoepithelial cells with abundant clear cytoplasm (2,3). However, literature describing the cytological features of EMCs is scarce. This might, partly, be attributed to the fact that establishing a diagnosis of EMC on the basis of cytomorphological features alone is often challenging owing to its variable cytological patterns and close morphologic mimics. This diagnostic difficulty is even more amplified at the metastatic sites, when the primary neoplasm is known. A search into the literature also reveals that often these neoplasms are misinterpreted as benign on cytology (4). Immunocytochemistry (IHC) performed on the cell-block can help in obtaining a definite cytologic diagnosis in such cases. Herein, we present a case of metastatic epithelial-myoepithelial carcinoma in a female, presenting with a neck mass and lytic lesion in the acetabulum, diagnosed by fine needle aspiration cytology (FNAC) and immunocytochemistry on the cell-block.

## CASE REPORT

**Figure 1:**
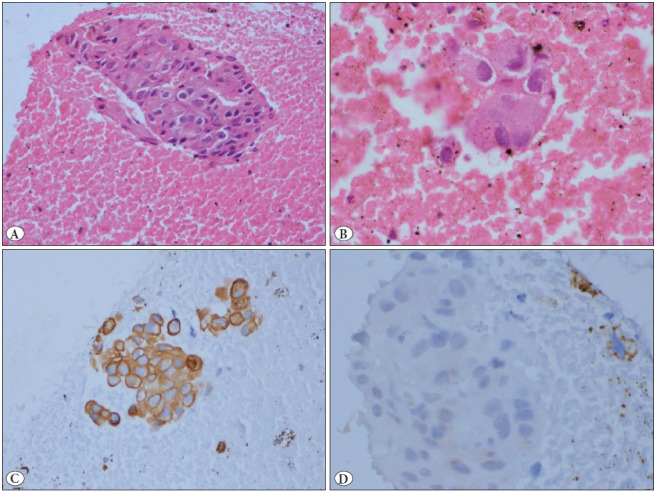
**A,B)** Section from the acetabular biopsy showing few sheets of tumor cells with mild to moderate nuclear pleomorphism, eccentric nuclei, coarse chromatin, occasional small nucleoli, and a moderate amount of cytoplasm (H&E; A:x20; B:x40). **C)** Immunohistochemistry for cytokeratin 7 showing strong diffuse positivity in the tumor cells (IHC; x20). **D)** Immunohistochemistry for p63 is negative in the tumor cells (IHC; x40).

A 58-year-old female presented with a history of low-backache for the last month, which was moderate in intensity and continuous in nature. On clinical examination, a single lymph node in the right lower cervical region (level III) measuring 2x2.5 cm in diameter, which was mobile, non-tender and firm in consistency was identified. According to the patient, the swelling was present for more than 20 years and was neither associated with pain nor an increase in size. There was no other significant lymphadenopathy and no sensory or motor neurological deficit. She had undergone surgery 35 years ago for a right-sided upper neck swelling, the medical records of which were not available. Radiological investigations revealed a lytic lesion in the left acetabulum. A computerised tomography (CT)-guided biopsy was obtained from the lesion. The histopathological examination of the hematoxylin and eosin (H&E) stained sections revealed occasional fragments of dead bone with a few clusters of round to polygonal tumor cells with mild cellular pleomorphism, eccentrically placed round nuclei, coarse chromatin, occasional prominent nucleoli, and a moderate amount of cytoplasm. Immunohistochemistry revealed the tumor cells to be positive for cytokeratin-7 (CK7) and GATA3 and negative for TTF1 and p63 (Figure 1). Based on the histopathological and immunohistochemical features, a diagnosis of metastatic carcinoma was rendered, with a suggestion of a primary of breast origin. Following this, the patient was worked-up for confirming the breast primary. However, clinical examination, mammography and positron emission tomography (PET) all failed to identify any lesion in the breast or lungs.

**Figure 2:**
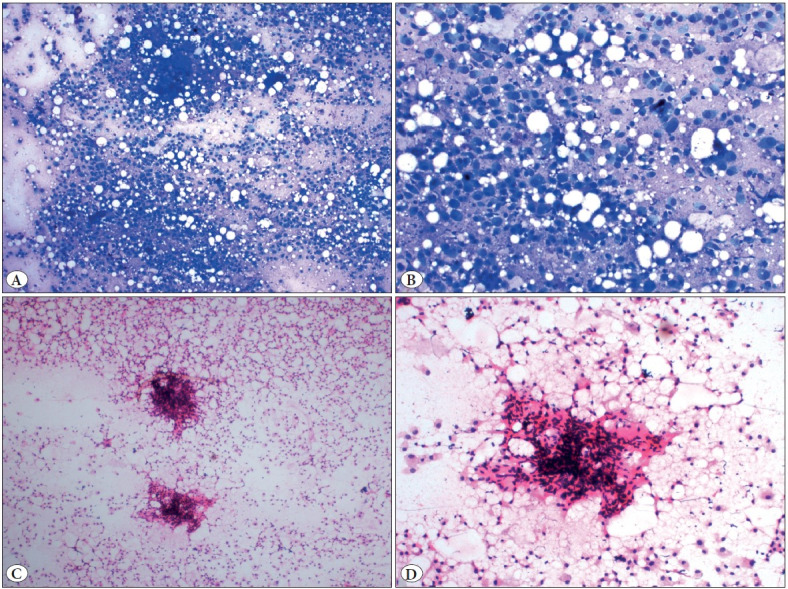
**A)** Cellular smear showing predominantly a dispersed population of round to polygonal tumor cells (MGG; x4). **B)** Smear showing mildly pleomorphic tumor cells with eccentrically placed round nucleus with fine chromatin and moderate to abundant amount of cytoplasm, representing the myoepithelial cells (MGG; x20). **C)** Smear showing scattered myoepithelial tumor cells with similar morphology and occasional cluster of smaller cells with scanty cytoplasm (H&E; x2). **D)** Higher magnification showing the myoepithelial cells with an occasional cluster of smaller cells along with a stromal fragment (H&E; x10).

**Figure 3:**
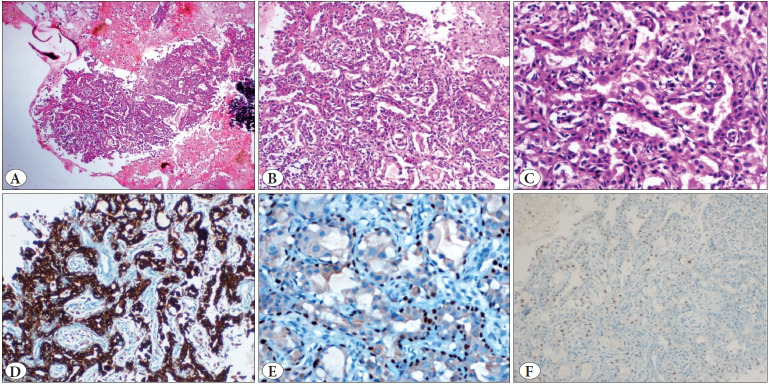
**A)** Section from the cell-block showing tumor cells arranged in a tubular pattern along with a fragment showing crushed lymphoid tissue (H&E; x2). **B,C)** Section showing the tubules lined by a dual layer of tumor cells with mild to moderate nuclear pleomorphism, the luminal cells with moderate cytoplasm and abluminal cells with tumor cells with scanty cytoplasm (H&E; B:x10; C:x20). **D)** CK7 shows strong diffuse membrano-cytoplasmic positivity in the luminal tumor cells (IHC; x10). **E)** p63 shows strong diffuse nuclear positivity in the abluminal tumor cells (IHC; x20). **F)** GATA3 shows nuclear positivity in scattered tumor cells (IHC; x4).

Consequently, FNAC from the right cervical swelling was advised and simultaneously a sample for cell-block preparation was also collected. The smears were cellular and showed tumor cells predominantly dispersed singly as well as arranged in loose clusters in a background of chondromyxoid stroma. The tumor cells were round to polygonal with mild pleomorphism, eccentrically placed round nuclei, coarse chromatin, occasional prominent nucleoli, and a moderate amount of cytoplasm with some showing cytoplasmic vacuolations. Additionally, occasional loose clusters of relatively smaller cells with scant cytoplasm were also noted (Figure 2). The H&E stained section from the cell-block revealed tumor cells arranged in the form of tubules lined by a dual layer of tumor cells. The luminal cells showed a scant to moderate amount of pale to eosinophilic cytoplasm and the basally placed cells showed a moderate amount of clear to vacuolated cytoplasm. A few scattered mitotic figures and occasional lymphoid aggregates were also noted. On performing IHC, the luminal cells showed strong diffuse membranous positivity for CK7 (epithelial marker) and the abluminal cells showed strong nuclear positivity for p63 (myoepithelial marker). Less than 10% of tumor cells showed nuclear positivity for GATA3 (Figure 3). Based on the cytomorphological features on smears and cell-block and the immunocytochemistry, a final diagnosis of metastatic epithelial-myoepithelial carcinoma was given.

## DISCUSSION

EMCs are low-grade malignant neoplasms with high frequency of recurrence, reaching close to 50% (3). Cytomorphological features of EMC of the salivary glands have been sporadically described in the literature (5–8). The identification of dual cell population comprising smaller ductal epithelial and larger myoepithelial cells in the smears is the major clue to the diagnosis. However, preponderance of either cell type in the smears is the major limitation in establishing an accurate cytodiagnosis. Rarely, the tumors may show marked nuclear pleomorphism, high mitotic activity, and necrosis. However, the majority of the cases demonstrate a deceptively innocuous cytomorphology, thereby compounding the diagnostic difficulty (4–9).

The major differential diagnoses on cytomorphology include pleomorphic adenoma, myoepithelioma and plasmacytoma, especially when the myoepithelial component predominates in the smears. Cell-block sections can provide useful information regarding the tumor cell architecture, as was evident in the index case. Additionally, immunohistochemistry can be performed on the cell-block sections for supplementing the morphologic diagnosis. A valuable learning point highlighted by the present report is that nuclear positivity for GATA3 should not always be recognized as an indicator of breast or urinary bladder origin. It is important to remember that variable GATA3 positivity can also be seen in some of the salivary gland neoplasms. Although amongst the salivary neoplasms, salivary duct carcinoma and mammary analogue secretory carcinoma show the most consistent immunostaining for GATA3 but the same can also be seen in EMCs (10,11). Hence the need to include salivary gland neoplasms in the list of differential diagnoses for cases with GATA3 positivity.

In conclusion, the present report, in addition to highlighting the cytomorphological and immunocytochemical features of EMC, reemphasizes the diagnostic utility of FNAC as a minimally invasive technique for diagnosis of such challenging cases.

## CONFLICT of INTEREST

The authors declare no conflict of interest.
